# Vascular dysfunction in obese diabetic db/db mice involves the interplay between aldosterone/mineralocorticoid receptor and Rho kinase signaling

**DOI:** 10.1038/s41598-018-21087-5

**Published:** 2018-02-13

**Authors:** Aurelie Nguyen Dinh Cat, Glaucia E. Callera, Malou Friederich-Persson, Ana Sanchez, Maria Gabriela Dulak-Lis, Sofia Tsiropoulou, Augusto C. Montezano, Ying He, Ana M. Briones, Frederic Jaisser, Rhian M. Touyz

**Affiliations:** 1 Institute of cardiovascular and medical sciences, University of Glasgow, Glasgow, United Kingdom; 2 Kidney Research Centre, Ottawa Hospital Research Institute, University of Ottawa, Ottawa, Canada; 3 Medical Cell Biology, Uppsala University, Uppsala, Sweden; 4 Departamento de Fisiología, Facultad de Farmacia, Universidad Complutense, Madrid, Spain; 5 Department of Pharmacology, School of Medicine, Universidad Autónoma de Madrid, CIBER de Enfermedades Cardiovasculares, Madrid, Spain; 6 INSERM 1138 Team 1, Centre de Recherche des Cordeliers, Paris, France

## Abstract

Activation of aldosterone/mineralocorticoid receptors (MR) has been implicated in vascular dysfunction of diabetes. Underlying mechanisms are elusive. Therefore, we investigated the role of Rho kinase (ROCK) in aldosterone/MR signaling and vascular dysfunction in a model of diabetes. Diabetic obese mice (db/db) and control counterparts (db/+) were treated with MR antagonist (MRA, potassium canrenoate, 30 mg/kg/day, 4 weeks) or ROCK inhibitor, fasudil (30 mg/kg/day, 3 weeks). Plasma aldosterone was increased in db/db versus db/+. This was associated with enhanced vascular MR signaling. Norepinephrine (NE)-induced contraction was increased in arteries from db/db mice. These responses were attenuated in mice treated with canrenoate or fasudil. Db/db mice displayed hypertrophic remodeling and increased arterial stiffness, improved by MR blockade. Vascular calcium sensitivity was similar between depolarized arteries from db/+ and db/db. Vascular hypercontractility in db/db mice was associated with increased myosin light chain phosphorylation and reduced expression of PKG-1α. Vascular RhoA/ROCK signaling and expression of pro-inflammatory and pro-fibrotic markers were exaggerated in db/db mice, effects that were attenuated by MRA. Fasudil, but not MRA, improved vascular insulin sensitivity in db/db mice, evidenced by normalization of Irs1 phosphorylation. Our data identify novel pathways involving MR-RhoA/ROCK-PKG-1 that underlie vascular dysfunction and injury in diabetic mice.

## Introduction

The high mortality and morbidity rates worldwide associated with obesity-related type 2 diabetes are attributed, in large part, to cardiovascular complications. Evidence suggests a link between hyperaldosteronism, obesity and hypertension, and clinical findings show a positive correlation between body weight and plasma aldosterone levels^1,2^. We and others demonstrated that aldosterone, through mineralocorticoid receptor (MR) activation, is involved in the regulation of structural and functional vascular changes, in particular in endothelial dysfunction and vascular hypercontractility, often associated with obesity-related type 2 diabetes and hypertension. MR blockade improves obesity-related vascular injuries, as well as insulin sensitivity^3–7^. Although plasma levels of aldosterone are elevated and expression of MR is increased in obesity and diabetes^3,8,9^ mechanisms by which aldosterone/MR signaling impact on vascular remodeling and function in these conditions remains unclear. We recently demonstrated that adipocyte MR activation influences vascular function and signaling through redox-dependent mechanisms and RhoA/Rho kinase (ROCK)^10^. Activation of the RhoA/ROCK signaling pathway plays an important role not only in enhanced vascular tone in hypertension^11–13^, but also in cellular metabolic processes, including insulin resistance^14–18^. It can be surmised therefore that the RhoA/ROCK pathway constitutes a potential point of cross-talk linking metabolic and hemodynamic abnormalities with insulin resistance and obesity. The role of ROCK as an effector of aldosterone/MR activation in vascular injury in obesity-associated diabetes is unknown and was the focus of our study.

## Results

### Morphological features of db/db and db/+ mice

**Figure 1:**
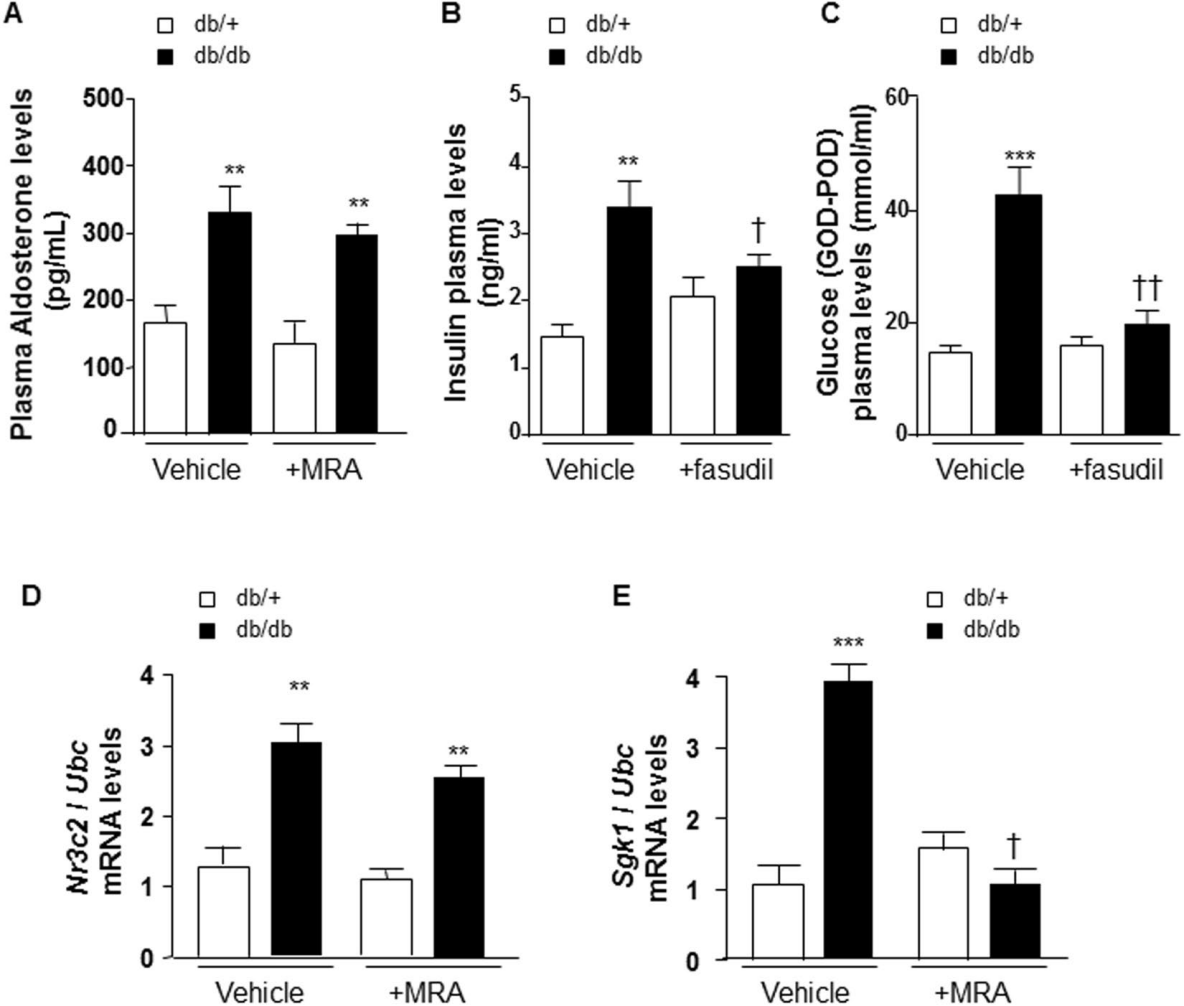
Plasma aldosterone, insulin and glucose concentrations and Nr3c2 and Sgk1 levels in arteries from db/db versus db/+ mice. (**A**) Plasma aldosterone levels are increased in db/db. MR antagonist treatment has no effect. Non fasting plasma levels of insulin (**B**) and glucose (**C**) are increased in db/db vs db/+ mice, and fasudil reduces both in db/db, but not in db/+. *Nr3c2* (**D**) and *Sgk1* **(E)** mRNA levels are increased in mesenteric arteries from db/db vs db/+ mice. MR antagonist treatment abolished increased *Sgk1*, but not *Nr3c2* mRNA levels in mesenteric arteries from db/db versus db/+ mice. *Ubc* was used as housekeeping gene for normalization. Results are expressed mean ± SEM, n = 6–8 mice per group, **p < 0.01, ***p < 0.001 db/db vs db/+, ^†^p < 0.05, ^††^p < 0.01 vehicle vs +MRA. MRA: MR antagonist (canrenoate); Nr3c2: nuclear receptor subfamily 3 group C member 2 (mineralocorticoid receptor); vs: versus. Vehicle: saline NaCl 0.9%; Ubc: ubiquitin C.

Body weight was greater in db/db mice versus db/+ (35.2 ± 0.6 vs 18.5 ± 0.4 g, n = 6 mice per group, p < 0.001) with no change in systolic blood pressure (data not shown). Fasting plasma glucose was increased in db/db versus db/+ (30.1 ± 3 vs 12.3 ± 6 mmol/ml, n = 6 mice per group, p < 0.01). Plasma aldosterone levels were increased in db/db compared with db/+ control mice (Fig. 1A). Canrenoate did not have any effect on plasma aldosterone levels (Fig. 1A). Interestingly, fasudil reduced non fasting plasma levels of insulin and glucose in db/db, but not in db/+ mice (Fig. 1B,C). Canrenoate treatment did not reduce plasma levels of insulin and glucose in db/db mice (Supplementary Fig. 1A,B).

### Upregulation of aldosterone/MR signaling in arteries from db/db mice

Transcripts levels of *Nr3c2* (MR gene) (Fig. 1D) and serum and glucocorticoid regulated kinase 1 (*Sgk-*1), a downstream target of MR activation (Fig. 1E) were increased in mesenteric arteries from db/db compared to db/+ control mice. Canrenoate treatment reduced the increase in vascular *Sgk1* mRNA levels (Fig. 1E), but not in vascular *Nr3c2* mRNA levels (Fig. 1D). Although protein levels of Nr3c2 were unchanged in all groups, protein levels of the plasminogen activator inhibitor-1 (PAI-1), known to be a downstream target of vascular MR activation, were significantly increased in db/db vs db/+ mice. Canrenoate treatment reduced this increase (Supplementary Fig. 1C,D).

### Effect of MR blockade with canrenoate on vascular structural and functional changes in db/db mice

**Figure 2:**
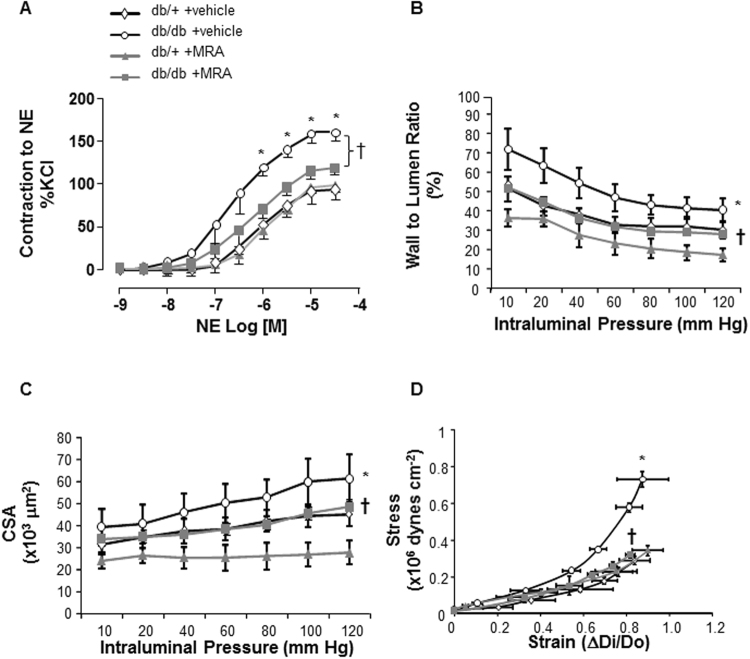
Vascular remodeling and function in arteries from db/db versus db/+ mice. (**A**) NE-induced contraction of mesenteric resistance arteries from control db/+ and db/db mice, treated by an MR antagonist (30 mg/kg/day, 4 weeks) or vehicle (saline), was performed using a wire myograph. Increased NE-induced contractility in db/db mice was prevented by canrenoate treatment. Structural and mechanical parameters were assessed by pressurized myography in mesenteric arteries from db/db vs db/+ mice. Vascular wall to lumen ratio (**B**) and CSA (**C**) were increased in db/db mice. (**D**) The stress-strain relationship was shifted to the left indicating that vascular stiffness is increased in db/db vs db/+ mice. All of these vascular changes were prevented by canrenoate treatment. Results are expressed mean ± SEM, n = 6–8 mice/group, *p < 0.05 db/db vs db/+, ^†^p < 0.05 vehicle vs +MRA. CSA: cross sectional area; MRA: MR antagonist (canrenoate). Vehicle: saline NaCl 0.9%.

Maximum responses to high concentration of potassium chloride (KCl) were identical between db/+ control and db/db mice (response to KCl: db/+ vs db/db, 2.0 ± 0.3 vs 1.9 ± 0.2 mN/mm; n = 6 mice per group, non significant (NS)). Norepinephrine (NE)-induced contraction of mesenteric arteries with intact endothelium was significantly increased in db/db versus db/+ control mice (Fig. 2A), as well as in arteries in which the endothelium had been mechanically denuded (Supplementary Fig. 2). Chronic treatment with potassium canrenoate (30 mg/kg/day, 4 weeks) prevented vascular hypercontractility to NE in arteries from db/db vs db/+ mice (Fig. 2A). The hypercontractility is not associated with changes in the active pressure which has been estimated with the myography data settings and was not significantly modified between groups (Supplementary Table S2).

Db/db mice displayed hypertrophic inward remodeling (Supplementary Fig. 3A–C) in small resistance arteries with increased vascular wall to lumen ratio and cross sectional area (CSA) in vehicle-treated obese db/db mice (Fig. 2B,C). These structural changes were prevented by canrenoate treatement. Moreover, the leftward shift of the stress-strain relationship, shown in Fig. 2D, indicated decreased elasticity in mesenteric arteries from db/db mice, which was prevented by canrenoate treatment.

### Rho kinase inhibitor fasudil improved vascular hypercontractility of arteries from db/db mice

**Figure 3:**
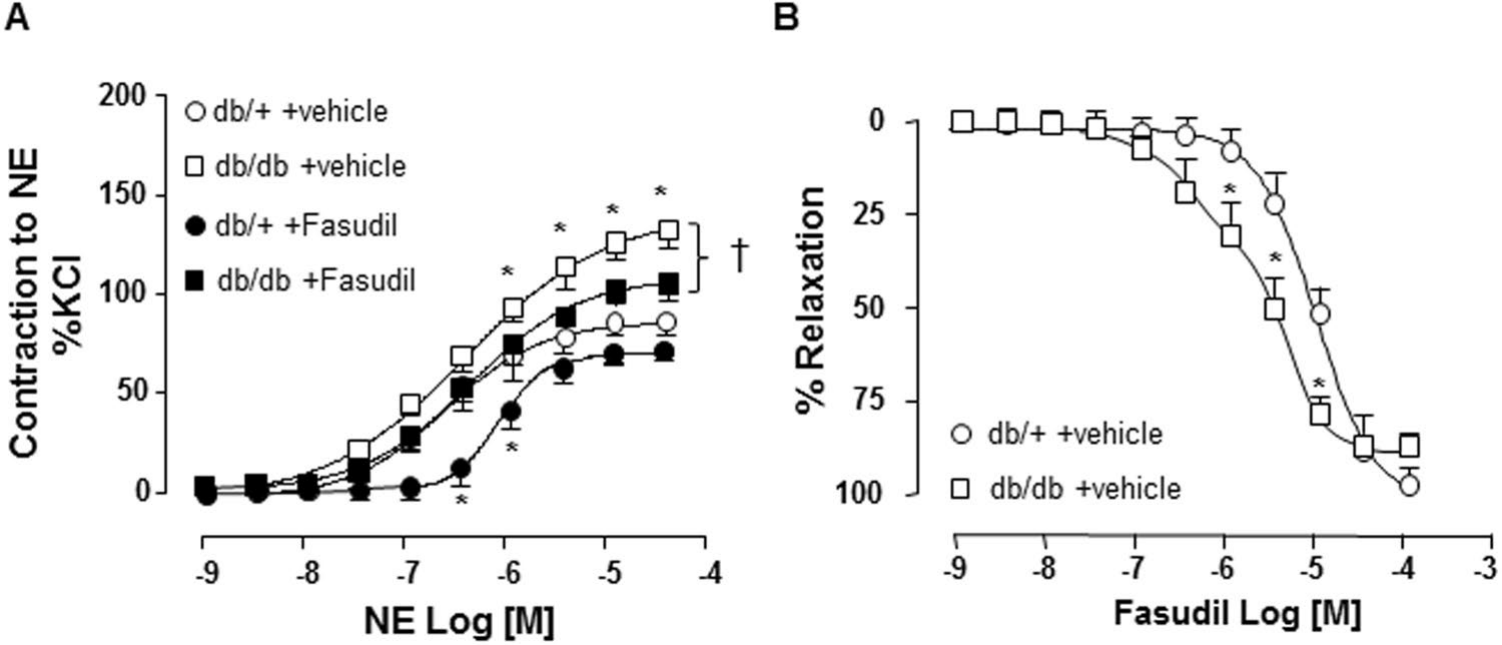
Rho kinase inhibitor fasudil improved contractility of arteries from db/db mice. (**A**) NE-induced contraction of mesenteric resistance arteries from control db/+ and db/db mice, treated by a Rho kinase inhibitor Fasudil (30 mg/kg/day, 3 weeks) or vehicle (saline), was performed using a wire myograph. (**B**) Dose-dependent fasudil-induced relaxation curve (10^−9^ to 10^−4^ mol/l) was shifted to the left for db/db vs db/+ mice. Data are presented as mean ± SEM; n = 6–8 mice/group, *p < 0.05 db/db vs db/+, ^†^p < 0.05 vehicle vs +fasudil. NE: norepinephrine; Vehicle: saline NaCl 0.9%.

Small GTPases of the Rho family influence vascular tone primarily by regulating smooth muscle contraction. Considering the significant alteration of vascular contractility in db/db mice, we explored the possibility that RhoA/ROCK may be involved. Db/db and db/+ mice were treated *in vivo* with fasudil, which decreased NE-induced contraction in mesenteric resistance arteries from both strains (Fig. 3A). Moreover, the relaxation curve obtained with increasing doses of fasudil was significantly shifted to left (Fig. 3B), indicating that arteries from db/db compared to db/+ mice are more sensitive to fasudil due to an exacerbation of ROCK activity in the vasculature of db/db mice.

### Vascular contractility regulated by ROCK is calcium-independent in db/db mice

ROCK activation is often associated with enhancement of calcium sensitivity. We hypothesized that arteries of db/db mice are more sensitive to calcium than arteries of db/+ controls, which may account partially for the hypercontractility observed in basal conditions. Calcium sensitization was assessed by stepwise increases of extracellular calcium concentration (0 to 5 mmol/l) in endothelium-denuded and depolarized (90 mmol/l KCl) mesenteric arteries. However, our results showed no alteration of the calcium sensitivity in db/db vs db/+ mice since there is no difference in contractile responses between arteries from db/db and db/+ mice in basal conditions, suggesting no alteration in calcium sensitivity (Supplementary Fig. 4A).

We next addressed whether ROCK contributes to vascular calcium sensitization by incubating arteries with fasudil (10 μmol/l, 30 min). Fasudil decreased calcium sensitivity similarly in db/+ and db/db mice (Supplementary Fig. 4B), but the gap is slightly greater in arteries from db/db (db/db vs db/+: −42.9 versus −27.4% of KCl 90 mmol/l), suggesting that ROCK is over-activated in arteries from db/db versus db/+ mice, but not calcium-independent. In fasudil-treated mice, calcium-induced contraction was decreased in arteries from db/db but not in db/+ mice (Supplementary Fig. 4C), which is in line with our previous *in vitro* results with fasudil.

### Rho kinase activity and signaling are increased in arteries from db/db mice

**Figure 4:**
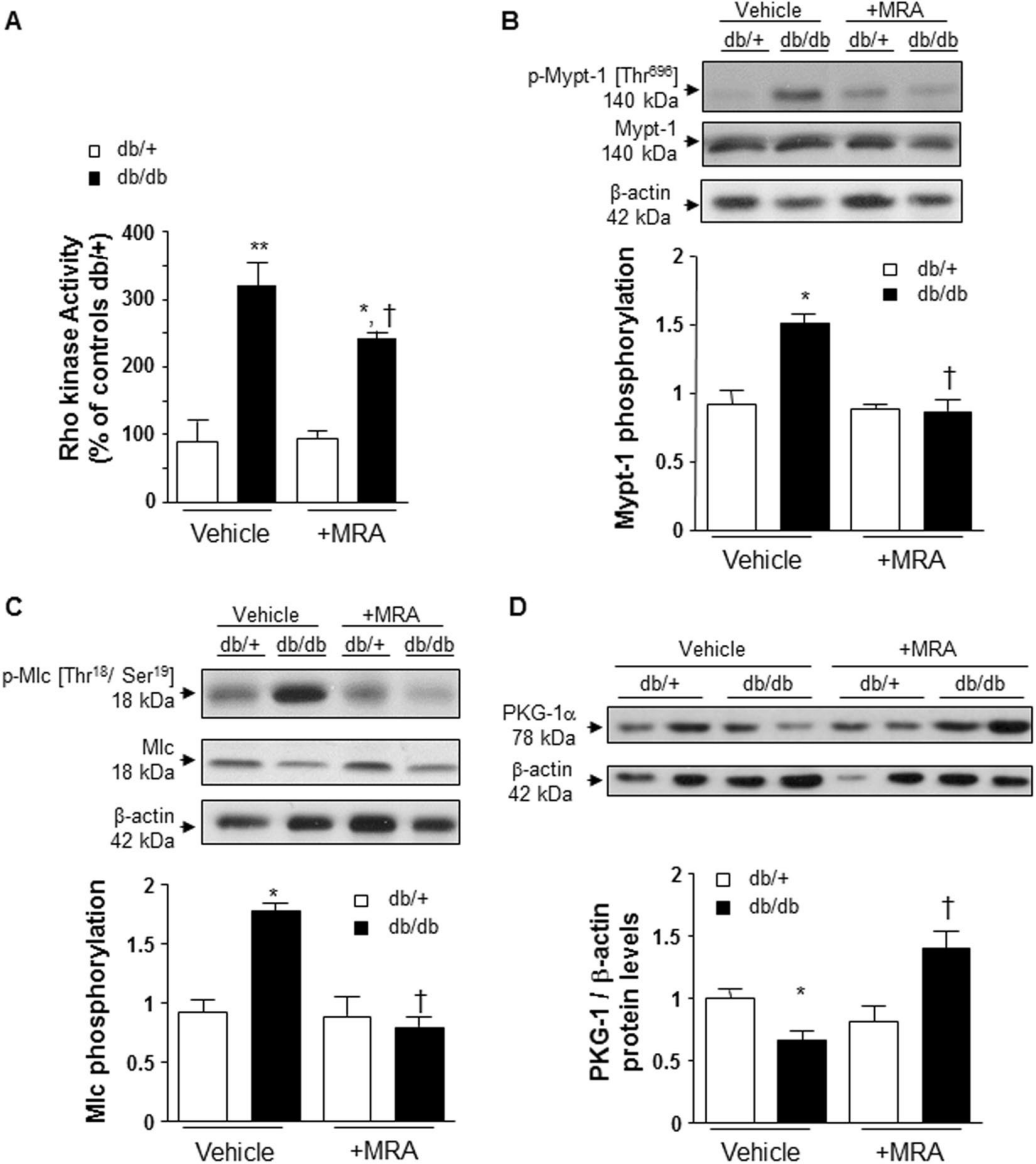
Rho kinase activity is increased in mesenteric arteries from db/db versus db/+ mice. (**A**) Rho kinase activity was assessed by enzymatic immunoassay. Upregulation of downstream targets of Rho kinase activation with Mypt-1 **(B)** and Mlc **(C)** phosphorylation in db/db vs db/+ mice. (**d**) PKG-1α protein levels are decreased in mesenteric arteries from db/db vs db/+ mice. These changes were prevented by canrenoate treatment. Results are expressed as mean ± SEM, n = 6–8 mice per group, *p < 0.05, **p < 0.01 db/db vs db/+, ^†^p < 0.05 vehicle vs +MRA. Vehicle: saline NaCl 0.9%.

ROCK activity was significantly increased (3.4-fold) in mesenteric arteries from db/db compared to db/+ mice (Fig. 4A). This was associated with increased phosphorylation of downstream targets of ROCK signaling, myosin phosphatase subunit 1 (Mypt1) (Fig. 4B) and myosin light chain (Mlc) (Fig. 4C). Fasudil significantly reduced the levels of Mypt1 and Mlc phosphorylations, as expected (Supplementary Table S3). To investigate some molecular mechanisms involved in the increased vascular ROCK activity in db/db mice, we focused on cyclic GMP-dependent kinase G (PKG)-1α which negatively regulates ROCK activity. As shown in Fig. 4D, protein levels of PKG-1α were significantly decreased in mesenteric arteries from db/db mice compared with db/+ controls.

### Partial regulation of ROCK activity by MR

Chronic treatment with canrenoate partially decreased vascular ROCK activity in db/db mice (Fig. 4A). The increase in Mypt1 and Mlc phosphorylation was blunted by MR blockade (Fig. 4B,C). In addition, canrenoate treatment significantly increased PKG-1α protein levels in arteries from db/db vs db/+ mice (Fig. 4D). However, vascular ROCK-1 and ROCK-2 protein lvels were unchanged in all groups (Supplementary Fig. 5A,B). Moreover, protein kinase C (PKC) is also a key player which when is activated contributes to vascular hypercontractility, independently to rho kinase. In arteries from db/db mice, PKC phosphorylation was significantly increased compared to db/+. This was partially prevented by MR blockade (Supplementary Fig. 5C). These results suggest that MR regulate several signalling pathways in order to control vascular contractility.

### Fasudil improved vascular insulin signaling in arteries from db/db mice

**Figure 5:**
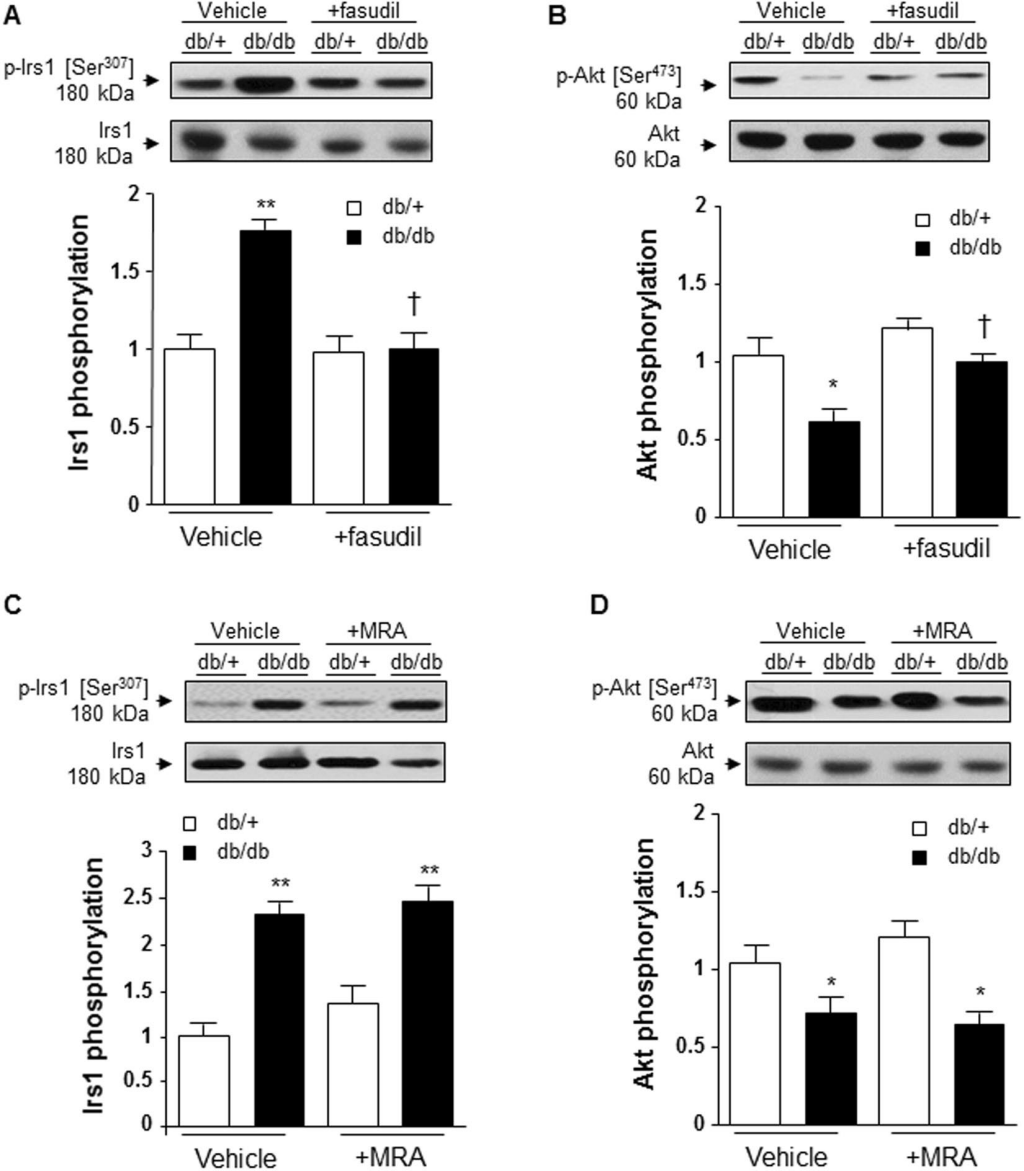
Fasudil improved vascular insulin signaling in db/db versus db/+ mice, and this is MR-independent pathway. Fasudil treatment prevents increased Ser-307-phosphorylation of Irs1 (**A**) and decreased Ser-473 phosphorylation of Akt (**B**) in mesenteric arteries from db/db vs db/+ mice. However, canrenoate treatment has no effect on the increase in Irs1 phosphorylation (**C**) neither on the decrease in Akt phosphorylation (**D**) in db/db mice. Data are presented as mean ± SEM; n = 6 to 8 mice/group. *p < 0.05, **p < 0.01 db/db vs db/+. Irs1: Insulin receptor substrate 1; MRA: MR antagonist canrenoate. Vehicle: saline NaCl 0.9%. Results are expressed mean ± SEM, n = 6–8 mice per group, *p < 0.05, **p < 0.01 db/db vs db/+, ^†^p < 0.05, ^††^p < 0.01 vehicle vs +fasudil. Irs1: insulin receptor substrate 1; Vehicle: saline NaCl 0.9%.

We next questioned whether fasudil influences insulin signaling in db/db mice and whether this involves MR. Previous studies examined the role of ROCK in insulin resistance in obesity^16–18^, but those studies focused on adipose tissue. Here we examined vascular insulin resistance. Insulin receptor substrate 1 (Irs1) phosphorylation at serine 307 (which negatively controls tyrosine-phosphorylated Irs1 by insulin receptor associated with insuline resistance) was significantly increased in mesenteric arteries from db/db mice (Fig. 5A). Akt activation, as evaluated through its phosphorylation on serine 473, was decreased in mesenteric arteries from db/db compared to db/+ mice (Fig. 5B). These results suggest that db/db mice display vascular insulin resistance. Fasudil significantly improved vascular insulin signaling and restored normal levels of Irs1 and Akt phosphorylation (Fig. 5A,B). This is in line with the decrease in plasma levels of insulin and glucose in db/db mice treated by fasudil, but not in db/+ mice. We next questioned whether the beneficial effects of fasudil on vascular insulin signaling was regulated by MR. We examined Irs1 and Akt phosphorylations in mesenteric arteries from db/db and db/+ mice, which were increased in db/db vs db/+ but canrenoate treatment had no effect (Fig. 5C,D). This is supported by the fact that plasma levels of insulin and glucose in canrenoate-treated db/db mice remained increased (Supplementary Fig. 1A,B).

### Effect of MR blockade on vascular levels of pro-fibrotic and pro-inflammatory markers in db/db mice

**Figure 6:**
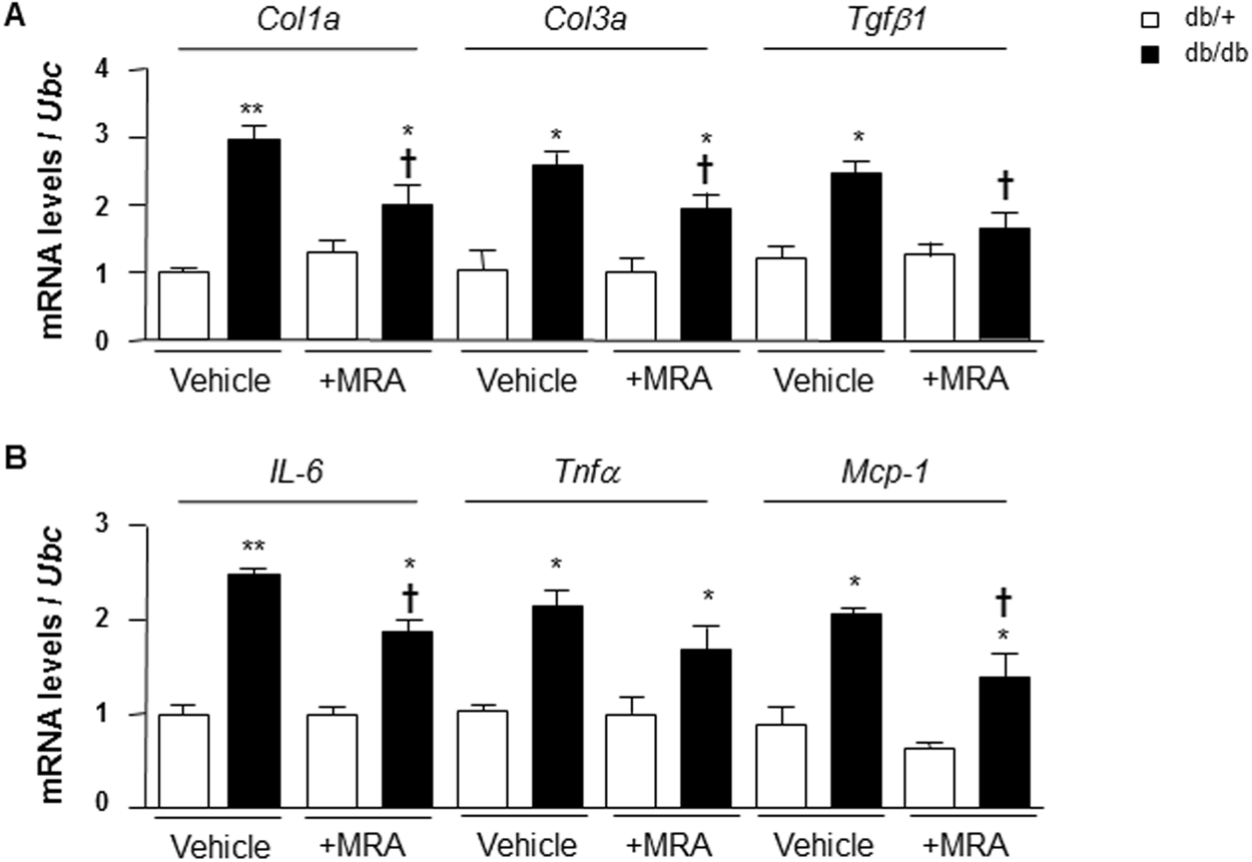
Pro-fibrotic and pro-inflammatory markers mRNA levels in arteries from db/db and db/+ mice. (**A**) Pro-fibrotic markers such as *Col1a*, *Col3a* and *Tgfb1* mRNA levels were increased in mesenteric arteries from db/db compared to db/+ mice. (**B**) Pro-inflammatory markers such as *IL-6*, *Tnfa* and *Mcp-1* mRNA levels were increased in mesenteric arteries from db/db compared to db/+ mice. *Ubc* was used as housekeeping gene for normalization. Results are expressed mean ± SEM, n = 6–8 mice per group, *p < 0.05, **p < 0.01 db/db vs db/+, ^†^p < 0.05 vehicle vs +MRA. Vehicle: saline NaCl 0.9%. IL-6: interleukine-6; Mcp-1: monocyte chemoattractant protein 1; Tnf*α*: tumor necrosis factor alpha; Tgfβ1: transforming growth factor beta 1; Ubc: ubiquitin C.

We analysed the expression of some pro-fibrotic and pro-inflammatory markers, which may be responsible for vascular damage in db/db mice and assessed whether MR plays a role. Vessels from db/db mice exhibited increased expression of *Col1a* (3-fold), *Col3a* (2.5-fold) and *Tgfβ1* (2.2-fold) compared to db/+ mice. These changes were partially reduced by canrenoate treatment (Fig. 6A). In addition, we evaluated mRNA levels of pro-inflammatory markers *Il-6*, *Tnfα* and *Mcp-1*. Gene expression of these pro-inflammatory markers was increased in db/db mice, effects that were attenuated by MRA (Fig. 6B).

## Discussion

**Figure 7:**
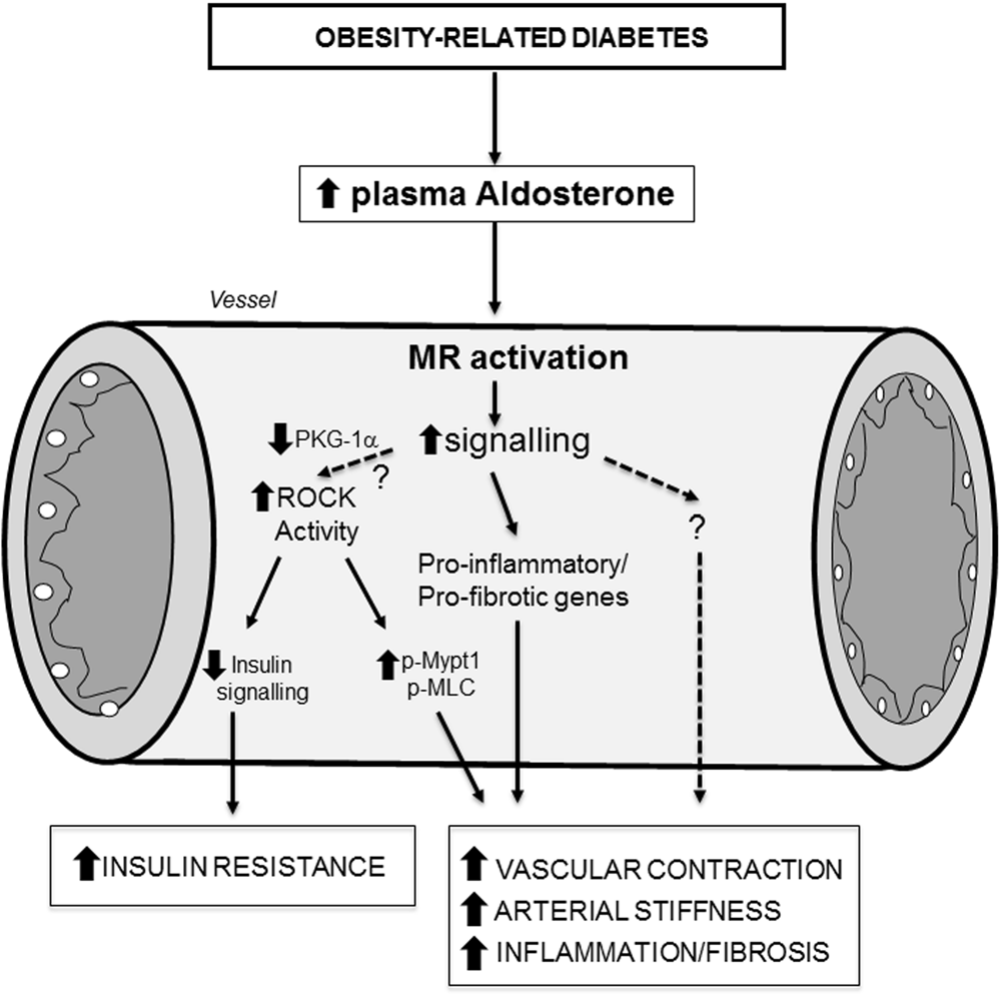
Hypothetical scheme of putative signaling mechanisms induced by aldosterone/MR over-activation in obesity. On one hand, aldosterone/MR induces ROCK activity through decreased PKG-1a, resulting in vascular hypercontractility of arteries from obese db/db mice. In obesity-related diabetes, ROCK may also contribute to vascular insulin resistance independently of MR. In addition, vascular remodeling, due to increased pro-fibrotic and pro-inflammatory signaling, is also regulated, in part, by MR. MLC: myosin light chain; Mypt1: myosin light chain phosphatase subunit 1; MR: mineralocorticoid receptor; PKG-1α: cGMP-dependent protein kinase G type 1 isoform alpha; ROCK: Rho kinase; p: phosphorylated.

Type 2 diabetes, a major risk factor for cardiovascular disease, is characterised by endothelial dysfunction and vascular remodeling, phenomena observed in experimental models and patients with diabetes. Exact mechanisms underlying these processes remain elusive, although oxidative stress, activation of the renin angiotensin system, advanced glycation end products (AGEs)/receptor for AGEs (RAGE) and hyperglycaemia have been implicated. We previously demonstrated an important role for aldosterone/MR in vascular damage and metabolic derangement in obesity-associated diabetes^5–7,10^ and others have shown a role for ROCK in endothelial dysfunction in cerebral arteries in diabetes^19^. Here we questioned the relationship between aldosterone/MR, ROCK and vascular dysfunction in a mouse model of type 2 diabetes. Major findings demonstrate that in db/db mice, which are obese and have hyperaldosteronism and vascular MR hyperactivation: (i) hypercontractility is reduced by canrenoate and fasudil, (ii) upregulation of vascular RhoA/ROCK pro-contractile signaling is ameliorated by ROCK inhibition and MR blockade and is related to decreased cGMP-dependent PKG-1 activity, (iii) increased vascular expression of pro-inflammatory and pro-fibrotic mediators are downregulated by canreonate, and (iv) ROCK contributes to insulin resistance through MR-independent mechanisms. Taken together our data suggest that hyperaldosteronism and MR hyperactivation in db/db mice are associated with vascular dysfunction and arterial remodeling through ROCK and PKG-1α signaling, which influences pro-contractile, pro-inflammatory and pro-fibrotic pathways. The downstream vascular signaling through the interplay between MR and ROCK is not a generalised phenomena, because insulin resistance appears to be ROCK-dependent and MR-independent. Our findings define a novel pathway through MR-ROCK in diabetes-associated vasculopathy, as summarised in Fig. 7.

It is generally accepted that obesity and diabetes are associated with an increased risk of hypertension, heart failure and atherosclerosis, but the mechanisms involved are still not fully understood. Adipose tissue is an endocrine organ with multiple metabolic roles in regulating whole-body physiology. Moreover, the fat surrounding the blood vessels, PVAT, plays a functional role in regulating the contractile state of the underlying smooth muscle cell layer by releasing vasoactive factors that diffuse into the vascular wall and modulates vascular tone^20–24^. In obesity and diabetes, PVAT is dysfunctional and induces vascular dysfunction, contributing to the development of cardiovascular disorders in these metabolic diseases^25–28^. We previously showed that PVAT influences vascular function and signaling through MR-dependent signaling pathways in obesity-associated diabetes^4,5,10^. Here, we performed our studies in the absence of PVAT and focused on MR-dependent signaling involved in vasoconstriction, vascular stiffness, fibrosis and inflammation. We highlight the critical role played by MR activation in these processes since canrenoate treatment normalised contractility and reduced vascular injurious events, including inflammation and fibrosis, in db/db mice. These vascular effects are independent of PVAT-derived factors.

We explored the potential role of RhoA/Rho kinase activation, a major signaling pathway regulating vascular smooth muscle contraction, to explain the hypercontractile phenotype and the vascular resistance observed in diabetic mice. Vascular insulin resistance is considered as an early contributor to vascular damage. The contractile state of vascular smooth muscle is driven by phosphorylation of the regulatory protein, MLC and reflects the balance of the Ca^2+^-calmodulin-dependent myosin light chain kinase and myosin light chain phosphatase (MLCP) activities^29^. Phosphorylation of Mypt1 at Thr-696 plays a dominant role in MLCP inhibition and has been shown to be increased in vasoconstriction-associated with hypertension^12,13^. Our molecular studies showed that ROCK activity and its downstream signaling (Mypt-1 and Mlc phosphorylations) are increased in arteries from db/db compared to db/+. Fasudil and canrenoate treatments restored vascular function in db/db mice, suggesting a role for MR upregulation and over-activation of ROCK/Mypt-1/Mlc signaling in hypercontractility in diabetes. MR blockade only partially decreased ROCK activity, suggesting that other mechanisms are also involved in MR-sensitive vascular dysfunction in diabetes, such as PKC activation and Ca^2+^-calcineurin pathways^30–33^. Indeed, our results showed that activation of PKC in db/db mice is also regulated by MR.

ROCK inhibition, but not MR blockade, improved insulin signaling by decreasing Irs1 phosphorylation at serine-307 and by increasing Akt phosphorylation. Serine-307 phosphorylation of Irs1 has been shown to mediate insulin resistance^34–36^. The fact that insulin sensitivity was unaffected by canrenoate, but was improved by fasudil indicates that vascular insulin resistance in db/db mice is ROCK-dependent and MR-independent. These results are different from other studies where MR blockade improved glucose tolerance and reduced insulin resistance in obesity^37–40^. However those studies were performed in experimental models of diet-induced obesity, whereas we examined obese db/db mice. Our findings unravel a novel ROCK mechanism underlying vascular insulin resistance in diabetic mice and highlighted the interest in analyzing the effects of combination treatment MRA with fasudil. We previously showed that MR over-activation in adipocytes (Adipo-MROE mice) leads to weight gain, insulin resistance and dyslipidaemia but also increased H_2_O_2_ levels associated with hypocontractility through activation of PKG-1α^10^.

In summary, our study demonstrates an important role for aldosterone/MR upregulation and ROCK hyperactivation in endothelium-dependent and -independent vascular hypercontractility and dysfunction in db/db mice. The interplay between MR and ROCK influences pro-contractile, pro-inflammatory and pro-fibrotic molecular processes that underlie diabetes-associated vascular changes. These phenomena are highly regulated because insulin resistance is ROCK-dependent and MR-independent. Our novel findings emphasize the functional importance of MR and ROCK activation in cardiovascular complications associated with obesity and diabetes. While canrenoate and fasudil, when given separately, ameliorated vascular damage in db/db, some responses were only partial. Combining MR blockers with ROCK inhibitors may have additive vasoprotective effects that might reduce cardiovascular complications in diabetes as reported by a clinical study demonstrating that eplerenone decreased the enhanced ROCK activity in circulating leukocytes in hypertensive patients^41^. Such a notion, which awaits further confirmation, may have therapeutic potential especially in patients with diabetes and high cardiovascular risk.

## Material and Methods

The study was approved by the Animal Ethics Committee of the University of Ottawa. All studies in animals were conducted in accordance with the National Institutes of Health Guide for the Care and Use of Laboratory Animals. All procedures were in accordance with institutional guidelines.

### Animal model

Male, 8 week-old, db/db mice [B6.BKS(D)-Lepr <db>/J], a model of type 2 diabetes mellitus, and age-matched heterozygote non-diabetic mice [(db/+)], were purchased from Jackson Laboratories (Maine, USA). Mice were maintained in the animal facility under controlled temperature (22–24 °C) and humidity, 12-hour light/dark cycles, fed with standard diet and water *ad libitum*.

Mice were treated with vehicle (saline), MR antagonist (MRA), potassium canrenoate for 4 weeks (30 mg/kg body weight/day) or Rho kinase inhibitor, fasudil (30 mg/kg body weight/day) for 3 weeks, by daily subcutaneous injections. At the end of the treatment, animals were euthanized by cervical dislocation and blood and tissues were collected. Mesenteric resistance arteries were used to assess vascular reactivity by wire myography and structural and mechanical properties by pressure myography. Biochemical and molecular biology studies were performed in plasma and vascular tissue.

### Vascular studie**s**

Second order branches of mesenteric arteries without perivascular fat (PVAT) were isolated from db/db and db/+ control mice and mounted on a wire myograph (DMT myograph, ADInstruments Ltd, Oxford, UK) as previously described^10^. Arteries were equilibrated for 20 min in physiological salt solution (in mmol/l: 120 NaCl, 25 NaHCO_3_, 4.7 KCl, 1.18 KH_2_PO_4_, 1.18 MgSO_4_, 2.5 CaCl_2_, 0.026 EDTA, and 5.5 glucose), continuously bubbled with 95% O_2_ and 5% CO_2_ to achieve a pH of 7.4 at 37 °C. Maximal responses with high potassium solution (120 mmol/l KCl) and 10^−5^ mol/l of norepinephrine (NE) were performed in order to verify functional integrity of the arteries. Contraction curves to cumulative increasing doses of NE (10^−9^ to 10^−5^ mol/l) were performed in arteries with and without (mechanically removed) intact endothelium. Dose-response curves for fasudil-induced relaxation (10^−9^ to 10^−4^ mol/l) were also obtained in arteries pre-contracted with NE at a concentration to achieve approximately 80% of maximal response.

Structural and mechanical parameters were evaluated with a pressure myograph (Living Systems, Burlington, Vermont). Briefly, the vessel was secured on two glass microcannulae, set to 70 mmHg and equilibrated (60 min, 37 °C) in oxygenated calcium-free Krebs–Henseleit solution (pH = 7.3–7.4; 0Ca-KHS, mmol/l: 119 NaCl, 4.7 KCl, 24 NaHCO_3_, 1.18 KH_2_PO_4_, 1.2 MgSO_4_ and 10 EGTA). The vessels were set to an internal pressure of 10 mmHg and a pressure–diameter curve was then obtained by increasing intraluminal pressure in 20 mmHg steps (from 10 to 120 mmHg), with internal and external diameters (D_i0Ca_ and D_e0Ca_, respectively) being measured. Vascular structural and mechanical parameters were calculated as previously described^10^.

Calcium sensitization was assessed by stepwise increases of extracellular calcium concentration (0 to 5 mmol/l) in endothelium-denuded and depolarized (90 mmol/l KCl) mesenteric arteries.

### Enzymatic immunoassays

#### Aldosterone levels measurement

Blood samples were collected into heparinized tubes, centrifuged at 2000 g at 4 °C for 15 min and plasma was further stored at −80 °C until analysis. Aldosterone concentrations were determined in plasma from db/db and db/+ control mice by enzyme immunoassay (EIA) (#10004377, Cayman Chemical, Ann Arbor, MI, USA) according to the manufacturer’s instructions.

#### Insulin and glucose levels determination

Non fasting insulin and glucose plasma levels were determined in db/db and db/+ control mice by ELISA kit (#EZRMI-13K, Merck Millipore, Fontenay sous bois, France) according to the manufacturer’s instructions and glucose GOD-POD kit (#981780, Thermo Fischer Scientific, Illkirch, France) optimized for Konelab analyzer (Konelab 20i, Thermo Fisher Scientific).

#### Rho Kinase Activity

Enzymatic activity of Rho kinase was evaluated using a Rho**-**associated Protein Kinase Activity Assay Kit (Merck Millipore #CSA001, East Midlands, United Kingdom) and the experiments were performed in mesenteric arteries and aortic protein lysates, according to the manufacturer’s instructions.

### Western blot analysis

Mesenteric arteries of db/+ and db/db mice were lysed for western blotting with buffer containing: 50 mmol/l Tris, pH 7.4; 5 mmol/l EGTA, 2.5 mmol/l EDTA, 1 mmol/l Na_2_VO_3_, 1 mmol/L PMSF, 1 µg/ml pepstatin A, 1 µg/ml leupeptin, and 1 µg/ml aprotinin and 0.5% Triton x-100. 30 μg of protein were separated by electrophoresis on a 10% SDS-polyacrylamide gel and transferred onto a nitrocellulose membrane (Bio-Rad, Hertfordshire, United Kingdom). Nonspecific binding sites were blocked with 5% skim milk in Tris-buffered saline solution with 0.1% Tween-20 for 1 hour at 24 °C. Membranes were then incubated with specific antibodies overnight at 4 °C. Antibodies were as follows: anti-Phospho-Akt (Ser-473) (1:1000, #9271, Cell Signalling Technology, Danvers, MA, USA), anti-Akt (1:1000, #9272), anti-Phospho-Irs1 (Ser-307) (1:1000, #2381, Cell Signalling Technology), anti-Irs1 (1:1000, #2382), anti-Phospho-Mypt1 (Thr-696) (1:1000, #sc-17556, Santa Cruz Biotechnology, CA, USA), anti-Mypt1 (H-130) (1:1000, #sc-25618), anti-Phospho-Mlc (Thr18/Ser19) (1:1000, #3674, Cell Signalling Technology), anti-Mlc (1:1000, #3672), anti-plasminogen activator inhibitor-1 (PAI-1) (1:1000, sc-6642, Santa Cruz Biotechnology), anti-phospho PKCβ/δ (Ser-660/Ser-662) (1:1000, sc-365463, Santa Cruz Biotechnology), anti-PKC (1:1000, sc-80, Santa Cruz Biotechnology), anti-PKG-1 (1:1000, 3248, Cell Signalling Technology), anti-Rock1 (1:1000, sc-17794, Santa Cruz Biotechnology) and anti-Rock-2 (1:1000, sc-398519, Santa Cruz Biotechnology). The Nr3c2 antibody (MR, 5D1, 1:500) was kindly provided by Pr C. Gomez-Sanchez. Membranes were incubated with horseradish peroxidase-conjugated secondary antibodies for 1 hour at room temperature. Thereafter signals were detected by chemiluminescence and visualised by autoradiography. The same membrane was used to determine β-actin expression as an internal housekeeping control using a mouse monoclonal antibody (1:10 000, Sigma Chemical Co, St Louis, MO, USA). Blots were analyzed densitometrically using the ImageJ software.

### Quantitative real time Polymerase Chain Reaction

Total RNA was extracted from mesenteric arteries as previously described^5^. Real time PCR was carried out on a 7900HT Fast Real-Time PCR machine with 384-Well Block Module (Applied Biosystems, Thermo Fischer Scientific, Paisley, United Kingdom) using gene-specific primers to quantify the relative abundance of each gene with SYBR Green I as the fluorescent molecule. The primers used were designed using the software Primer 3 and are listed in Electronic Supplemental Material Methods (Supplementary Table S1). PCR was performed in duplicate for each sample using a PCR Master mix for SYBR Green I (Applied Biosystems) containing 300 nmol/l primers, and 3 µl template cDNA in 10 µl total volume. PCR conditions consisted of an activation step of the *Taq* DNA polymerase (95 °C) for 10 min, followed by 40 cycles of 10 s at 95 °C (denaturation step) and 1 min at 60 °C (primer annealing, extension, and fluorescence acquisition). Serial dilution of pooled cDNA was used in each experiment to assess PCR efficiency. *Ubiquitin C* (*Ubc*) housekeeping gene was used as the reference gene for normalization. The relative copies number of the target genes were calculated with the 2^(−ΔΔCt)^ method, after assessment that PCR efficiency was 100%.

### Drugs and solutions

NE and potassium canrenoate were obtained from Sigma-Aldrich Ltd (Dorset, United Kingdom). Fasudil was obtained from Tocris (Cederlane Corp., Burlington, ON, Canada). NE was dissolved in distilled water. Fasudil was dissolved in dimethylsulfoxide. Potassium canrenoate was dissolved in sodium chloride NaCl 0.9% (saline).

### Data analysis

Values reported are means ± SEM. Differences between groups were assessed with the nonparametric Mann-Whitney test. Western blot and real time PCR data were statistically analysed with one-way ANOVA, followed by Bonferroni multiple comparison test. Vascular reactivity results were assessed with two-ways ANOVA with repeated measures, followed by Bonferroni multiple comparison test, as appropriate. Values of *p* < 0.05 were considered significant.

## Electronic supplementary material

Supplemental material

Supplemental dataset

## References

[1] Goodfriend TL, Kelley DE, Goodpaster BH, Winters SJ (1999). Visceral obesity and insulin resistance are associated with plasma aldosterone levels in women. Obes Res..

[2] Rossi GP (2008). Primary Aldosteronism Prevalence in hYpertension Study Investigators. Body mass index predicts plasma aldosterone concentrations in overweight-obese primary hypertensive patients. J Clin Endocrinol Metab..

[3] Guo C (2008). Mineralocorticoid receptor blockade reverses obesity-related changes in expression of adiponectin, peroxisome proliferator-activated receptor-gamma, and proinflammatory adipokines. Circulation..

[4] Nguyen Dinh Cat A (2011). Adipocyte-derived factors regulate vascular smooth muscle cells through mineralocorticoid and glucocorticoid receptors. Hypertension..

[5] Briones AM (2012). Adipocytes produce aldosterone through calcineurin-dependent signalling pathways: implications in diabetes mellitus-associated obesity and vascular dysfunction. Hypertension..

[6] Silva, M.A. *et al*. Spironolactone treatment attenuates vascular dysfunction in type 2 diabetic mice by decreasing oxidative stress and restoring NO/GC signalling. *Front Physiol*. **6**, 29, 10.3389/fphys.2015.00269. eCollection2015 (2015) 10.3389/fphys.2015.00269 PMC4593519 26500555

[7] Silva MA (2015). Mineralocorticoid receptor blockade prevents vascular remodelling in a rodent model of type 2 diabetes mellitus. Clin Sci (Lond)..

[8] Urbanet R (2015). Adipocyte Mineralocorticoid Receptor Activation Leads to Metabolic Syndrome and Induction of Prostaglandin D2 Synthase. Hypertension..

[9] Hirata A (2012). Contribution of glucocorticoid-mineralocorticoid receptor pathway on the obesity-related adipocyte dysfunction. Biochem Biophys Res Commun..

[10] Nguyen Dinh Cat A (2016). Adipocyte-Specific Mineralocorticoid Receptor Overexpression in Mice Is Associated With Metabolic Syndrome and Vascular Dysfunction: Role of Redox-Sensitive PKG-1 and Rho Kinase. Diabetes..

[11] Loirand G, Guilluy C, Pacaud P (2006). Regulation of Rho proteins by phosphorylation in the cardiovascular system. Trends Cardiovasc Med..

[12] Seko T (2003). Activation of RhoA and inhibition of myosin phosphatase as important components in hypertension in vascular smooth muscle. Circ Res..

[13] Guilluy C (2005). Inhibition of RhoA/Rho kinase pathway is involved in the beneficial effect of sildenafil on pulmonary hypertension. Br J Pharmacol..

[14] Kanda T (2006). Rho-kinase as a molecular target for insulin resistance and hypertension. FASEB J..

[15] Bach LA (2008). Rho kinase inhibition: a new approach for treating diabetic nephropathy?. Diabetes..

[16] Hara Y (2011). Rho and Rho-kinase activity in adipocytes contributes to a vicious cycle in obesity that may involve mechanical stretch. Sci Signal.

[17] Tokuyama H (2012). Role of mineralocorticoid receptor/Rho/Rho-kinase pathway in obesity-related renal injury. Int J Obes (Lond)..

[18] Noda, K. *et al*. Rho-kinase inhibition ameliorates metabolic disorders through activation of AMPK pathway in mice. *PLoS One*. **9**(11), e110446, 10.1371/journal.pone.0110446. eCollection2014 (2014). 10.1371/journal.pone.0110446 PMC4217731 25365359

[19] Didion SP, Lynch CM, Baumbach GL, Faraci FM (2005). Impaired endothelium-dependent responses and enhanced influence of Rho-kinase in cerebral arterioles in type II diabetes. Stroke..

[20] Eringa EC, Bakker W, van Hinsbergh VW (2012). Paracrine regulation of vascular tone, inflammation and insulin sensitivity by perivascular adipose tissue. Vascul Pharmacol..

[21] Lee HY, Després JP, Koh KK (2013). Perivascular adipose tissue in the pathogenesis of cardiovascular disease. Atherosclerosis..

[22] Brown NK (2014). Perivascular adipose tissue in vascular function and disease: a review of current research and animal models. Arterioscler Thromb Vasc Biol..

[23] Oriowo MA (2015). Perivascular adipose tissue, vascular reactivity and hypertension. Med Princ Pract..

[24] Even SE, Dulak-Lis MG, Touyz RM, Nguyen Dinh Cat A (2014). Crosstalk between adipose tissue and blood vessels in cardiometabolic syndrome: implication of steroid hormone receptors (MR/GR). Horm Mol Biol Clin Investig..

[25] Fernández-Alfonso MS (2013). Mechanisms of perivascular adipose tissue dysfunction in obesity. Int J Endocrinol..

[26] Szasz T, Bomfim GF, Webb RC (2013). The influence of perivascular adipose tissue on vascular homeostasis. Vasc Health Risk Manag..

[27] Chang L, Milton H, Eitzman DT, Chen YE (2013). Paradoxical roles of perivascular adipose tissue in atherosclerosis and hypertension. Circ J..

[28] Gollasch M (2017). Adipose-Vascular Coupling and Potential Therapeutics. Annu Rev Pharmacol Toxicol..

[29] Kimura K (1996). Regulation of myosin phosphatase by Rho and Rho-associated kinase (Rho-kinase). Science..

[30] Kizub IV (2014). Protein kinase C in enhanced vascular tone in diabetes mellitus. Int J Cardiol..

[31] Galmiche G (2014). Smooth muscle cell mineralocorticoid receptors are mandatory for aldosterone-salt to induce vascular stiffness. Hypertension..

[32] Tarjus A (2015). Role of smooth muscle cell mineralocorticoid receptor in vascular tone. Pflugers Arch..

[33] Amador CA (2016). Deletion of mineralocorticoid receptors in smooth muscle cells blunts renal vascular resistance following acute cyclosporine administration. Kidney Int..

[34] Hirosumi J (2002). A central role for JNK in obesity and insulin resistance. Nature..

[35] Sugita M, Sugita H, Kaneki M (2004). Increased insulin receptor substrate 1 serine phosphorylation and stress-activated protein kinase/c-Jun N-terminal kinase activation associated with vascular insulin resistance in spontaneously hypertensive rats. Hypertension..

[36] Aguirre V (2002). Phosphorylation of Ser307 in insulin receptor substrate-1 blocks interactions with the insulin receptor and inhibits insulin action. J Biol Chem..

[37] Hirata A (2009). Blockade of mineralocorticoid receptor reverses adipocyte dysfunction and insulin resistance in obese mice. Cardiovasc Res..

[38] Schäfer N (2013). Endothelial mineralocorticoid receptor activation mediates endothelial dysfunction in diet-induced obesity. Eur Heart J..

[39] Sherajee SJ (2012). Aldosterone induces vascular insulin resistance by increasing insulin-like growth factor-1 receptor and hybrid receptor. Arterioscler Thromb Vasc Biol..

[40] Bruder-Nascimento T, da Silva MA, Tostes RC (2014). The involvement of aldosterone on vascular insulin resistance: implications in obesity and type 2diabetes. Diabetol Metab Syndr..

[41] Fujimura N (2012). Mineralocorticoid receptor blocker eplerenone improves endothelial function and inhibits Rho-associated kinase activity in patients with hypertension. Clin Pharmacol Ther..

