# Supplementary material for: Vascular dysfunction in obese diabetic db/db mice involves the interplay between aldosterone/mineralocorticoid receptor and Rho kinase signaling
Source: Sci Rep. 2018 Feb 13;8:2952. doi: 10.1038/s41598-018-21087-5 (PMC5811612; doi:10.1038/s41598-018-21087-5)

SUPPLEMENT MATERIAL

Vascular dysfunction in obese diabetic db/db mice involves the interplay between aldosterone/mineralocorticoid receptor and Rho kinase signalling.

Aurelie NGUYEN DINH CAT[1], Glaucia E. CALLERA[2], Malou FRIEDERICH-PERSSON[1,3], Ana SANCHEZ[4], Maria DULAK-LIS[1], Sofia TSIROPOULOU[1], Augusto C. MONTEZANO[1], Ying HE[2], Ana M. BRIONES[5], Frederic JAISSER[6], Rhian M. TOUYZ[1,2].

[1] Institute of cardiovascular and medical sciences, University of Glasgow, Glasgow, United Kingdom; [2] Kidney Research Centre, Ottawa Hospital Research Institute, University of Ottawa, Ottawa, Canada; [3] Medical Cell Biology, Uppsala University, Uppsala, Sweden; [4] Departamento de Fisiología, Facultad de Farmacia, Universidad Complutense, Madrid, Spain; [5] Department of Pharmacology, School of Medicine, Universidad Autónoma de Madrid, Spain; [6] INSERM 1138 Team 1, Centre de Recherche des Cordeliers, Paris, France.

Running title: MR-ROCK interplay in vascular dysfunction.

Correspondence:

Aurelie Nguyen Dinh Cat, PhD
Institute of Cardiovascular & Medical Sciences
BHF Glasgow Cardiovascular Research Centre
University of Glasgow
126 University Place
Glasgow G12 8TA
Tel: + 44 (0)141-330-8015
Fax: + 44 (0)141-330-3360
Email: cattuong.ndc@gmail.com

Supplementary Table 1. Primers for real-time PCR analysis.

Genes	Forward Primer	Reverse Primer
m-*Ubc*	GGTCAAACAGGAAGACAGACGTA	CACACCCAAGAACAAGCACA
m-*Nr3c2*	TCACATTTTTAACATGTGACGGC	TCCTTTTCACCAGCAAGCT
t-*Nr3c2*	GGCTACCACAGTCTCCCTGA	CGTTGACAATCTCCATGT
m-*Sgk1*	GATGGGCCTGAACGATTTTA	GGACCCAGGTTGATTTGTTG
m-*Il-6*	TCTAATTCATATCTTCAACCAAGAG	TGGTCCTTAGCCACTCCTTC
m-*Tnf-α*	TGCCTATGTCTCAGCCTCTTC	GAGGCCATTTGGGAACTTCT
m-*Mcp1*	CCCACTCACCTGCTGCTACT	TCTGGACCCATTCCTTCTTG
m-*Tgfβ1*	GTCCTTGCCCTCTACAACCA	GTTGGACAACTGCTCCACCT

Ubc, ubiquitin C; Nr3c2, nuclear receptor subfamily 3, group C member 2 (MR, mineralocorticoid receptor); Sgk1, serum glucocorticoid-induced kinase 1; Il-6: interleukine-6; Tnf-α: tumor necrosis factor alpha; Mcp1: monocyte chemoattractant protein 1; Tgfβ1: transforming growth factor beta 1; m, mouse; t, total: endogenous and exogenous MR.

Supplementary Table 2. Effective active pressure in phosphorylations in db/db vs db/+ control mice.

	db/+ + vehicle	db/+ +MRA	db/db +vehicle	db/db +MRA
**Normalized lumen diameter (µm)**	190.3 ± 18	202 ± 12.7	227.2 ± 23.4	213.5 ± 19.7
**Wall Tension (mN/mm)**	3.3 ± 0.7	2.1 ± 0.4	3.0 ± 0.7	2.6 ± 0.6
**Active effective pressure (mmHg)**	257.4 ± 51.7	160 ± 24.9	203.7 ± 24.4	204.6 ± 33.7

The effective normalized lumen diameters (l1) (µm) of the mesenteric arteries of each animal in each group, calculated with the internal circumference (obtained after normalization procedure) divided by π;

The active wall tension T (mN/mm) which is given by the force response per unit vessel length = F/(2 x segment length);

The active effective pressure (mmHg) is an estimated pressure required to expand the vessel to the measured internal circumference P=T/(l1/2). It takes into account the normalized lumen diameter as well as the active wall tension.

Supplementary Table 3. Effect of fasudil on Mypt1 and Mlc phosphorylations in db/db vs db/+ control mice.

	db/+ + vehicle	db/+ +fasudil	db/db +vehicle	db/db +fasudil
**p-Mypt1**	1.02 ± 0.05	0.85 ± 0.18	2.92 ± 0.41	1.25 ± 0.22
**p-Mlc**	1.16 ± 0.32	1.05 ± 0.2	2.35 ± 0.2	0.92 ± 0.13

Mypt1 and Mlc phosphorylations were normalized with total. Mypt1, myosin light chain phosphatase subunit 1; Mlc, myosin light chain; p-, phosphorylated

Supplementary legends.

Supplementary Fig. 1. Effect of MR blockade on plasma levels of insulin and glucose, Nr3c2 and PAI-1 protein levels in arteries from db/db versus db/+ mice. Canrenoate treatment has no effect in insulin **(A)** nor glucose **(B)** plasma levels in db/db vs db/+ mice. **(C)** Nr3c2 protein levels are unchanged in all groups. **(D)** PAI-1 (protein levels are increased in mesenteric arteries from db/db vs db/+ mice. MR antagonist treatment reduced increased PAI-1 in mesenteric arteries from db/db versus db/+ mice. β-actin was used as housekeeping protein for normalization. Results are expressed mean ± SEM, n=6-8 mice per group, ** p<0.01 db/db vs db/+, † p<0.05 vehicle vs +MRA. MRA: MR antagonist (canrenoate); Nr3c2: nuclear receptor subfamily 3 group C member 2 (mineralocorticoid receptor); vs: versus. PAI-1: plasminogen activator inhibitor-1.

Supplementary Fig. 2. Vascular hypercontractility in db/db vs db/+ control mice. Contractile responses to cumulative and increasing doses of norepinephrine (NE, 10^{-9} to 10^{-5} mol/l) in mesenteric arteries without intact endothelium were significantly increased in arteries from db/db vs db/+ mice. Data are presented as mean ± SEM; n=6 to 8 mice/group. * p<0.05 db/db vs db/+. KCl: potassium chloride; NE: norepinephrine. Vehicle: saline NaCl 0.9%.

Supplementary Fig. 3. Vascular remodeling in arteries from db/db vs db/+ mice. Structural and mechanical parameters were assessed by pressurized myography in mesenteric arteries from obese db/db vs lean db/+ mice. (A) Internal (lumen) diameter. **(B)** External diameter. **(C)** Wall thickness. Results are expressed mean ± SEM, n=6-8 mice/group, * p<0.05 db/db vs db/+, † p<0.05 vs Vehicle. MRA: MR antagonist (canrenoate); Vehicle: saline NaCl 0.9%.

Supplementary Fig. 4. ROCK activation in calcium sensitization. (A) Calcium sensitization was assessed by contractile responses to increasing doses of extracellular calcium, from 0–5 mmol/l (expressed as log values of molar concentrations of $CaCl_2$) in endothelium-denuded and depolarized (90 mmol/l K^+) mesenteric arteries of db/+ and db/db mice. In basal conditions, there is no difference in $CaCl_2$-induced contraction between db/+ and db/db mice. **(B)** Fasudil (10^{-6} mmol/l, added 30 min prior to dose-response curve) decreased calcium sensitivity in db/+ and db/db (closed symbols), $CaCl_2$-induced contractions were decreased in fasudil-treated db/db mice (right panel) as well as in vehicle-treated db/+ mice (left panel). **(C)** Similar results were obtained with chronic treatment with fasudil (30 mg/kg/day, 3 weeks; closed black symbols) of db/+ (left panel) and db/db mice (right panel). Results are expressed mean ± SEM, n=6 mice/group, † p<0.05 vs Vehicle. Vehicle: saline NaCl 0.9%.

Supplementary Fig. 5. Vascular ROCK-1 and ROCK-2 protein levels and PKC activation in db/db and db/+ mice. Protein levels of vascular ROCK-1 **(A)** and ROCK-2 **(B)**

are unchanged between db/db and db/+ mice. Canrenoate treatment has no effect. **(C)** PKC phosphorylation is upregulated in db/db vs db/+ mice. This was partially prevented by canrenoate treatment. Data are presented as mean ± SEM; n=6 to 8 mice/group. * $p<0.05$, ** $p<0.01$ db/db vs db/+, † $p<0.05$ vehicle vs +MRA. Vehicle: saline NaCl 0.9%.. ROCK: rho kinase; MRA: MR antagonist canrenoate; PKC: protein kinase C; Vehicle: saline NaCl 0.9%.

Supplementary Fig. 1.

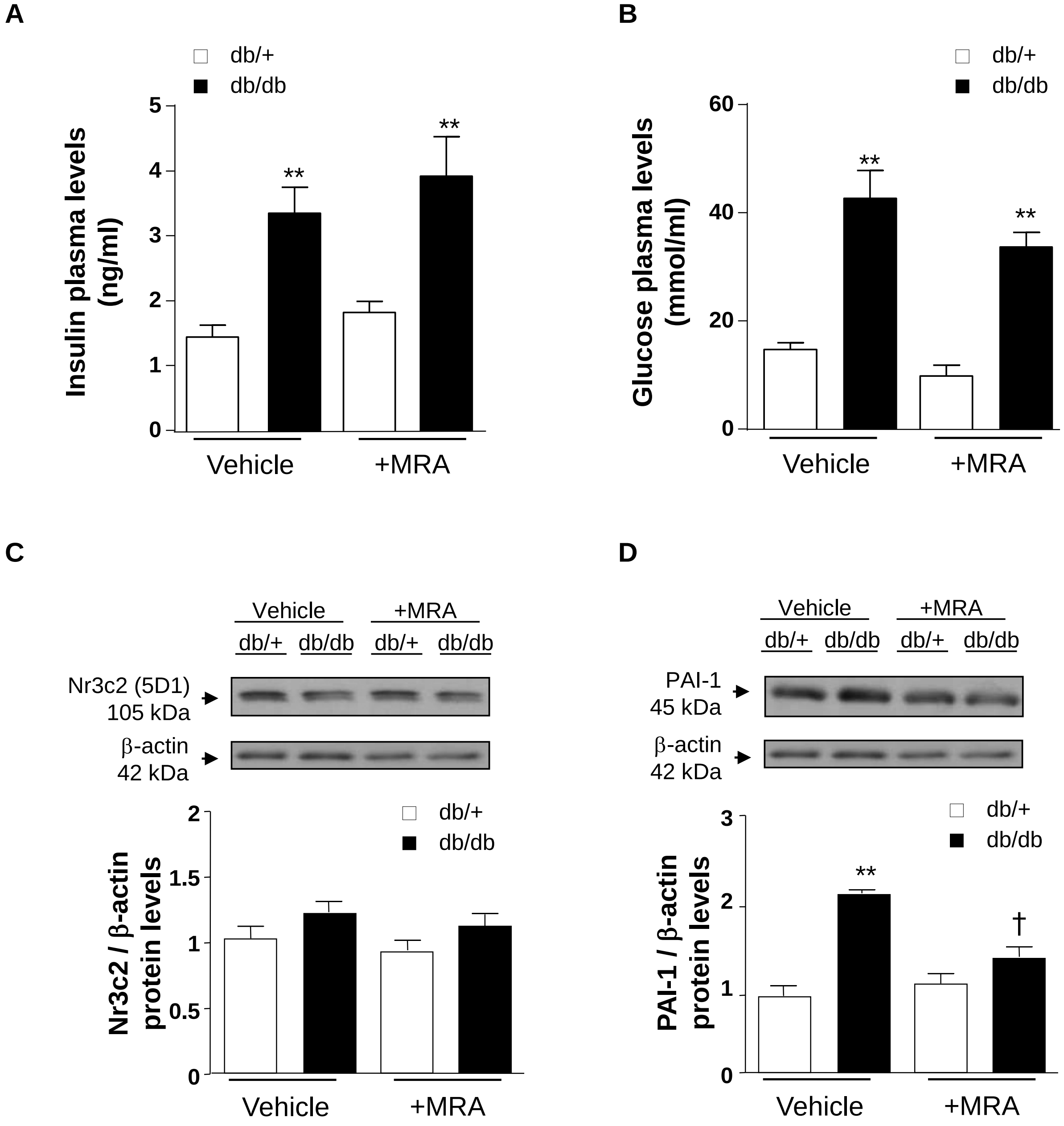

Supplementary Fig. 2.

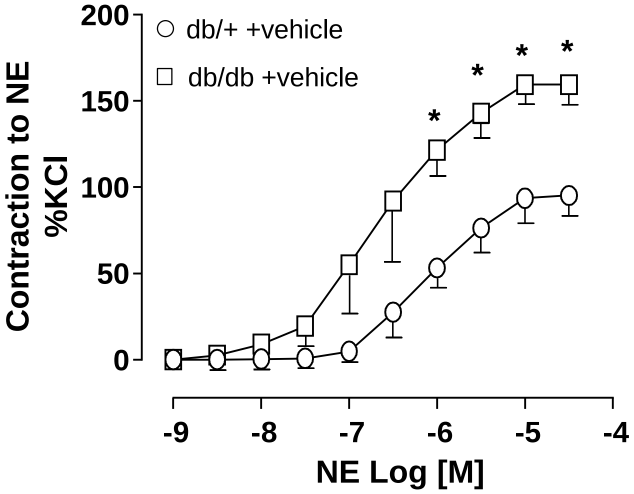

Supplementary Fig. 3.

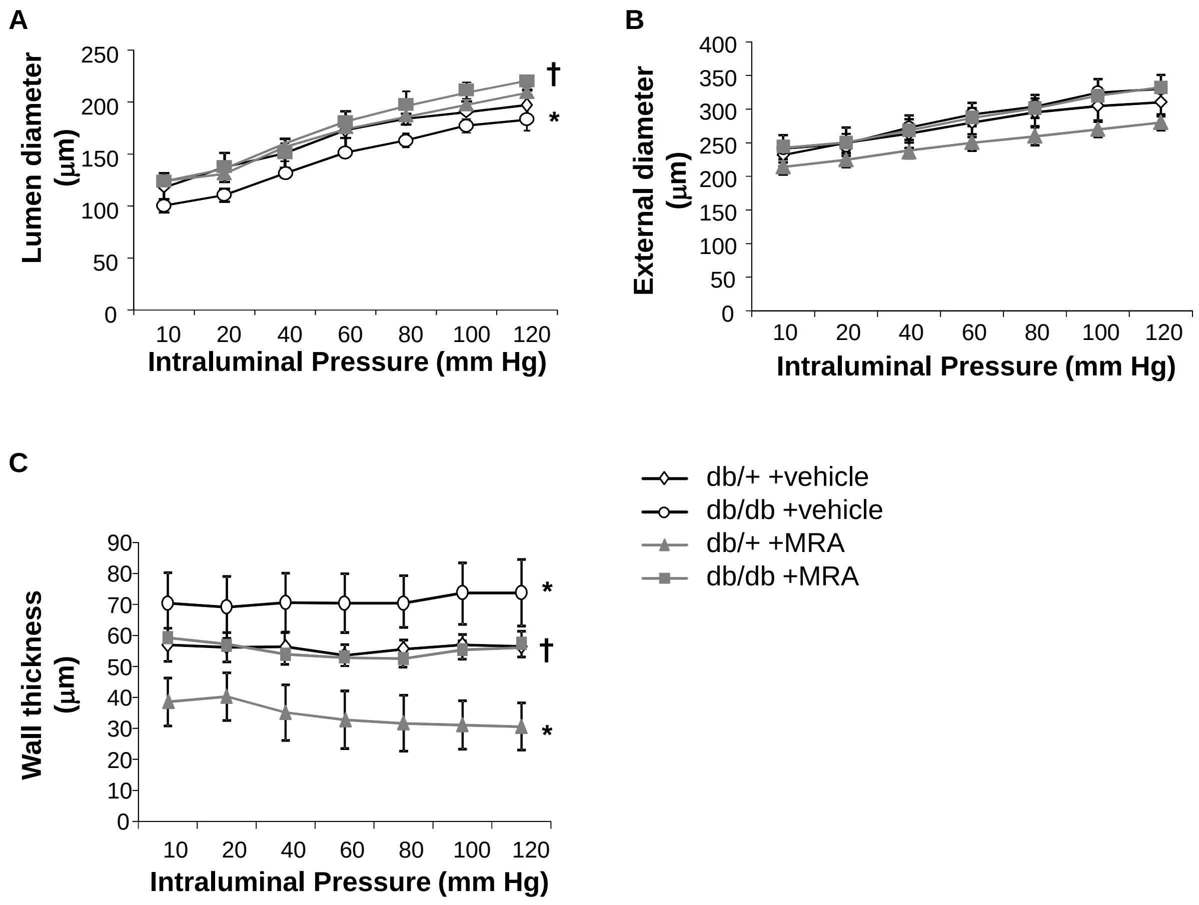

Supplementary Fig. 4.

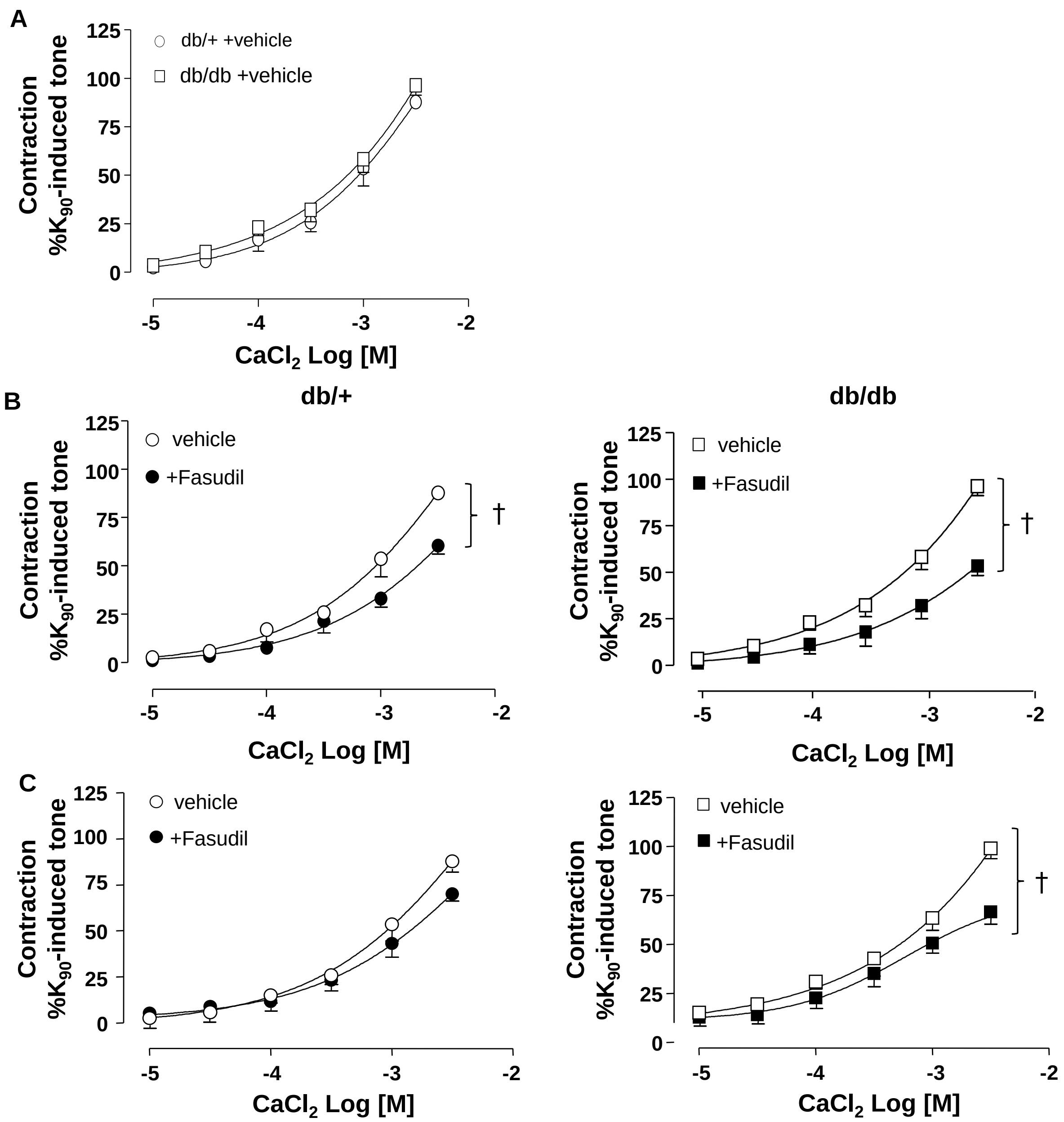

Supplementary Fig. 5.

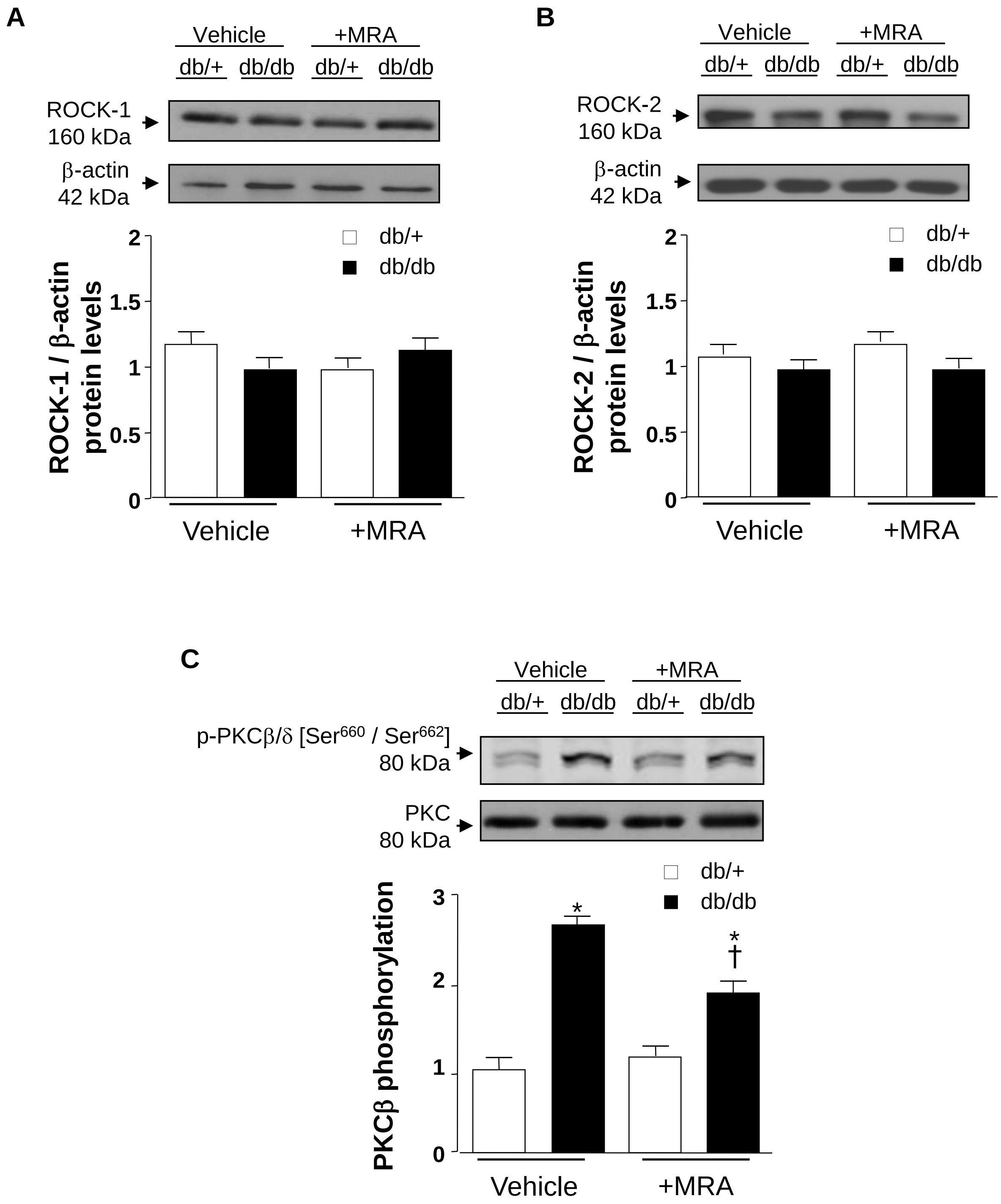

Supplement: Supplementary file 1 — Supplemental material [file 41598_2018_21087_MOESM1_ESM.pdf]
