# Supplementary material for: Vascular dysfunction in obese diabetic db/db mice involves the interplay between aldosterone/mineralocorticoid receptor and Rho kinase signaling
Source: Sci Rep. 2018 Feb 13;8:2952. doi: 10.1038/s41598-018-21087-5 (PMC5811612; doi:10.1038/s41598-018-21087-5)
Supplement: Supplementary file 2 — Supplemental dataset [file 41598_2018_21087_MOESM2_ESM.doc]

**SUPPLEMENTARY DATASET**

**Vascular dysfunction in obese diabetic db/db mice involves the interplay between aldosterone/mineralocorticoid receptor and Rho kinase signaling.**

Aurelie NGUYEN DINH CAT^1^, Glaucia E. CALLERA^2^, Malou FRIEDERICH-PERSSON^1,3^, Ana SANCHEZ^4^, Maria DULAK-LIS^1^, Sofia TSIROPOULOU^1^, Augusto C. MONTEZANO^1^, Ying HE^2^, Ana M. BRIONES^5^, Frederic JAISSER^6^, Rhian M. TOUYZ^1,2^.

^1^ Institute of cardiovascular and medical sciences, University of Glasgow, Glasgow, United Kingdom; ^2^ Kidney Research Centre, Ottawa Hospital Research Institute, University of Ottawa, Ottawa, Canada; ^3^ Medical Cell Biology, Uppsala University, Uppsala, Sweden; ^4^ Departamento de Fisiología, Facultad de Farmacia, Universidad Complutense, Madrid, Spain; ^5^ Department of Pharmacology, School of Medicine, Universidad Autónoma de Madrid, Spain; ^6^ INSERM 1138 Team 1, Centre de Recherche des Cordeliers, Paris, France.
